# Range Dynamics of Spongy Moth (*Lymantria dispar* L.) in Northern European Russia over the Past Two Centuries

**DOI:** 10.3390/insects16121189

**Published:** 2025-11-22

**Authors:** Andrey Selikhovkin, Nikita Mamaev, Ludmila Sherbakova, Sergey Sinev, Andrey Broshkov, Aleksandr Alekseev, Vyacheslav Martemyanov

**Affiliations:** 1 Department of Forest Protection, Wood Science and Game Management, Saint Petersburg State Forest Technical University, Institutski Lane 5, St. Petersburg 194021, Russia; 2 Zoological Institute of Russian Academy of Science, Universitetskaya Emb., 1, St. Petersburg 199034, Russia; 3 Department of Naturel Sciences, Novosibirsk State University, Pirogova Str. 2, Novosibirsk 630090, Russia; 4 Department of Forest Inventory, Management and Geographical Information Systems Saint Petersburg State Forest Technical University, Institutski Lane 5, St. Petersburg 194021, Russia; 5 Laboratory of Ecological Physiology, Institute of Systematics and Ecology of Animals, Siberian Branch of the Russian Academy of Sciences, Frunze Str. 11, Novosibirsk 630091, Russia; 6 Research Center for Genetics and Life Sciences, Sirius University of Science and Technology, Olympic Prospect 1, Sochi 353340, Russia

**Keywords:** nun moth, spongy moth, range expansion, climate change, pheromone trapping, invasive pest, population dynamics

## Abstract

Climate dynamics on our planet drive range border movements for many species. Insects, being ectothermic animals, significantly respond to changes in these conditions. We recently demonstrated northward expansion for important invasive pest species such as *Lymantria dispar* in central Asia. In the current study, we combined historical data and our modern data, using very sensitive pheromone trapping, to estimate the dynamics of the northern range border of *Lymantria dispar.*

## Simple Summary

Climate change drives many biological processes including population dynamics and range border movement for many species. In our study we analyzed both the literature and our original data of *Lymantria dispar* spreading within northern Europe over the last two centuries. *Lymantria dispar* is among the 100 most important invasive species on our planet. Thus, it is extremely important to observe the dynamics and direction of this species’ expansion to new territory. We demonstrate that in northern European regions there is no northward movement of the *Lymantria dispar* border range in a northward direction, which is opposite to the pattern observed in Asian populations of the same pest species.

## 1. Introduction

One potential threat associated with climate warming is increased heat availability for the development of forest insect pests in the northern part of their range. This creates conditions for the expansion of their ranges northward and the emergence of outbreaks of some species [1,2,3]. An increase in the frequency and intensity of outbreaks of woody plant pest reproduction has been noted in the northwest of the European part of Russia and Scandinavia. In particular, this is characteristic of wood-feeding insects and especially for the eight-toothed spruce bark beetle *Ips typographus* (Linnaeus, 1758) (Coleoptera: Scolytidae). An increase in outbreak frequency and the northward advancement of outbreak activity of the spruce bark beetle has been noted in Scandinavia [4,5], in the Leningrad oblast, and Karelia [6]. The secondary range of a species new to the fauna of Russia—the small spruce bark beetle *Ips amitinus* (Eichhoff, 1871) (Coleoptera: Scolytidae)—has rapidly expanded. Its range has already reached the Kola Peninsula [7]. An increase in outbreak activity has been noted in the northern part of the ranges for extremely dangerous species of folivorous insects—the Siberian silk moth *Dendrolimus sibiricus* (Tschetverikov, 1908) (Lepidoptera: Lasiocampidae) and the spongy moth *Lymantria dispar* (Linnaeus, 1758) (Lepidoptera: Erebidae) [8,9,10,11]. The northward advancement of spongy moth populations in the Asian part of Russia has been demonstrated [12]. In Scandinavia and in the northwestern regions of the European part of Russia, an increase in the frequency of outbreaks of winter moth *Operophtera brumata* (Linnaeus, 1758) and autumnal moth *Epirrita autumnata* (Borkhausen, 1794) (Lepidoptera: Geometridae) and a number of other pests of the assimilatory apparatus of woody plants has been noted [6,13,14,15].

Nevertheless, the connection between climate warming and both range expansion and increased outbreak activity is not always obvious. Some studies have shown the absence of dependence of range dynamics and frequency of pest outbreaks on the increase in degree-days [2,16]. Moreover, in a number of regions, particularly in the European part of Russia, a sharp decrease in the frequency of reproduction outbreaks of free-living phyllophagous insects has been noted. In the regions of the Northwestern Federal District, excluding the Kaliningrad oblast, typical pest species such as the pine beauty moth *Panolis flammea* (Denis et Schiffermüller, 1775) (Noctuidae), the buff-tip *Phalera bucephala* (Linnaeus, 1758) (Notodontidae), the vapourer *Orgyia antiqua* (Linnaeus, 1758) and the white satin moth *Leucoma salicis* (Staudinger, 1892) (Erebidae), as well as the bordered white *Bupalus piniaria* (Linnaeus, 1758) (Geometridae), have not produced reproduction outbreaks for 30 or more years. Previously, reproduction outbreaks of these pests were recorded at intervals of 10–15 years [6,9].

One of the most dangerous herbivores, and apparently the most well-known, is the spongy moth *Lymantria dispar*. Thousands of studies have been devoted to this species. The description of the spongy moth is present in all textbooks on forest and agricultural entomology and corresponding reference books [2,17,18,19,20], and its invasion of the North American continent has become a canonical example of the danger of the introduction of species to new regions [21] and placed it among the top 100 most dangerous invasive species on our planet. Mass reproduction outbreaks of the spongy moth in the territory of Russia have been recorded since the first half of the 19th century. In the Lesnoy Zhurnal (Russian Forestry Journal) and some other publications of the 19th and first decade of the 20th century, numerous reports of spongy moth reproduction outbreaks in the southern and central regions of Russia are provided. In the monograph by Prof. M. Kulagin [22] of Moscow Agricultural University, the governorates where the spongy moth was noted are listed.

Due to the absence of information on the current range boundary of the spongy moth in northwestern Russia (northeastern Europe) for the last 50 or more years, as well as the pronounced expansion northward in the Asian part of the continent [23], we aimed to assess the distribution of the spongy moth in the northern part of the European continent over the last two centuries, create maps based on both historical data and our actual data, and compare this data with climate dynamics in the North European region.

## 2. Materials and Methods

### 2.1. Historical Data Analysis

To create the map of the spread of *L. dispar* in northern Eastern Europe, we analyzed the detailed review of [22,24,25,26,27,28,29,30,31,32,33,34,35,36,37,38,39,40], who collected the data of *L. dispar* outbreaks from the 1840s to 1950s. Additionally, we used collections of insects in the Zoological Institute of the Russian Academy of Sciences and Saint Petersburg State Forest Technical.

### 2.2. Current Monitoring of the Northern Range Border of L. dispar in Europe

**Figure 1:**
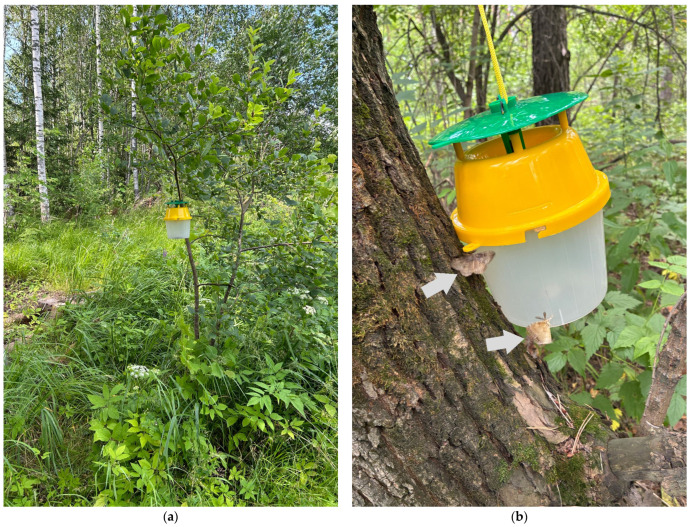
(**a**) Pheromone trap located in studied area (locality 5, 59°12′25.4″ N 29°58′47.1″ E; Horticultural Non-commercial Partnership Lutinovka, Divenskaya village, Leningrad oblast) (photo by L.N. Shcherbakova, 2023) and (**b**) trap with trace of disparlure (without dispenser); the picture was taken 5 min after placing the trap on the tree in the forest with a low population density of *Lymantria dispar* (53°19′17.8″ N 84°11′50.3″ E). Arrows demonstrate attracted males.

**Figure 2:**
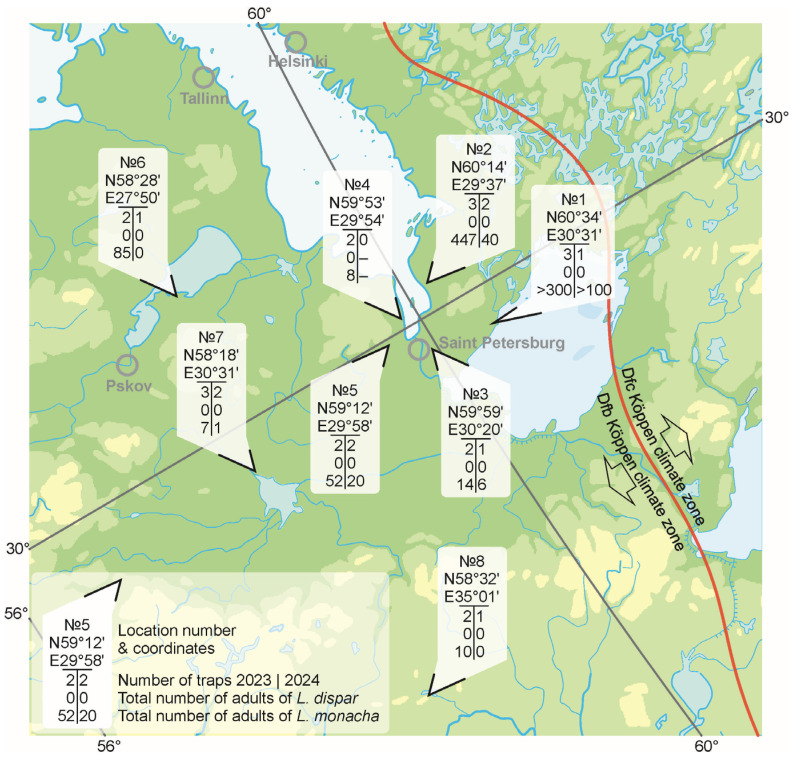
Distribution of pheromone traps across the territory of Leningrad, Novgorod, and Pskov oblasts and average number of *L. dispar* and *L. monacha* moths caught per trap in 2023/2024. Climate zone boundaries are indicated according to Kottek et al. [42].

To monitor the actual range border, we used the most sensitive approach—pheromone trapping—which works very well in low-population-density locations [23]. Plastic Unitrap traps were used [41] (Figure 1). Trapping was conducted from 15 July to 15 September in 2023 and 2024. Traps were placed in the Leningrad oblast and St. Petersburg, as well as in the northern part of the Pskov and Novgorod oblasts (Figure 2, Table 1). In 2023, two traps were installed in the State Museum-Reserve Peterhof (SMR Peterhof) and two traps in the territory of the park of the St. Petersburg State Forestry Academy. In 2024, trapping was not conducted in this territory.

Dispensers with spongy moth pheromones were purchased from the All-Russian Plant Quarantine Center (Bykovo, Russia) (in 2023) and from Trace incorporated (Boise, ID, USA), lot #88860300 (in 2024). To test the stability of disparlure produced by different producers, the following year after field use, we used the traps again (i.e., in 2025) but without pheromone dispensers. In other words, the trap contained only traces of disparlure. We placed these traps in the area of known *L. dispar* range (53°19′17.8″ N 84°11′50.3″ E) with low population density (no visible defoliation occurred in June 2025). To demonstrate the phenomenal sensitivity of males to disparlure, we present the picture taken five minutes after the trap was place in the forest (Figure 1b).

### 2.3. Identification of Collected Specimens

**Figure 3:**
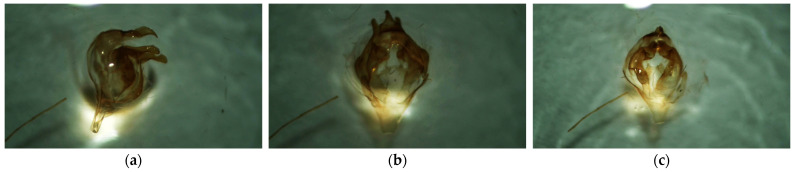
Male genitalia of nun moth *L. monacha* in different views: (**a**) lateral projection; (**b**) dorsal projection; (**c**) ventral projection (photo by N.A. Mamaev, 2024).

Material from traps was collected after completion of trapping. Most of the collected material could not be identified by external morphological features, since the survey was conducted at the end of the season. Therefore, identification of moths caught in pheromone traps was carried out by genitalia. Genitalia were boiled in 10% potassium hydroxide solution [43,44]. For comparison, genitalia of male specimens of *L. dispar* and *L. monacha* from the museum collection of SPbSFTU were used (Figure 3).

### 2.4. Temperature and Precipitation Dynamics in Eurasia over the Last Century

To assess climate dynamics in the studied region we (i) used maps demonstrating dynamics of outside temperature dynamics and precipitation dynamics; (ii) compared the average rate of both temperature and precipitation at 60 parallel (current northern border of *L. dispar* in Asia) in Europe and Asia.

**Figure 4:**
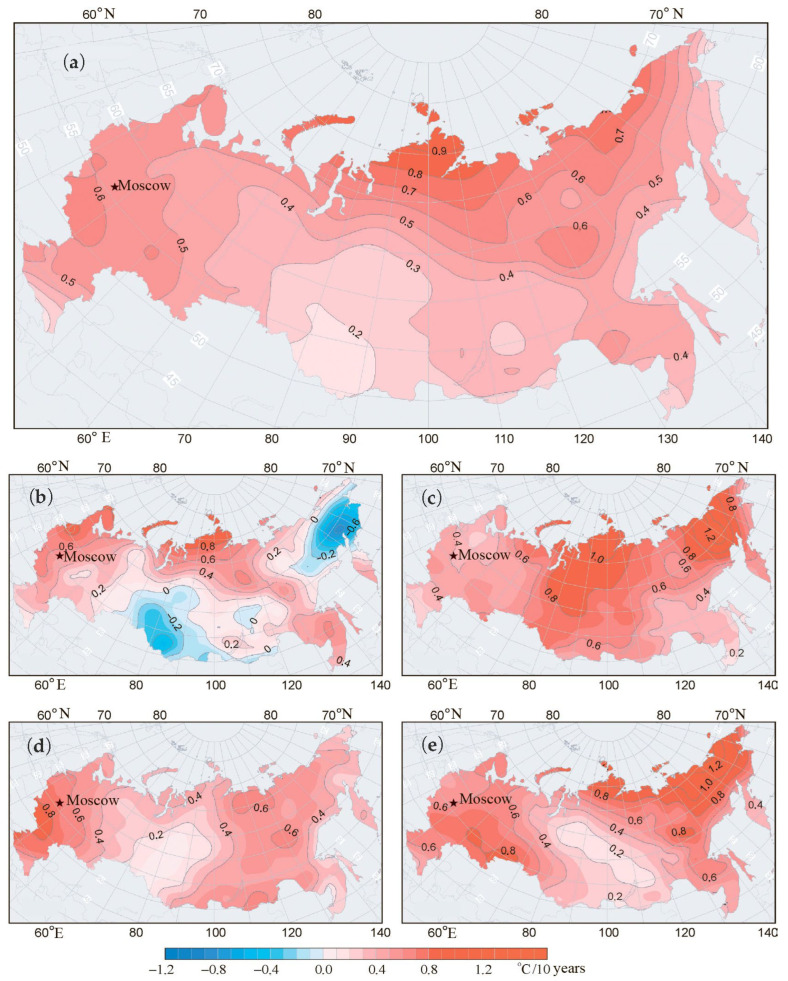
Spatial pattern of mean temperature variation in Russia for the years 1976–2012: (**a**) annual; (**b**) winter; (**c**) summer; (**d**) spring; (**e**) autumn. Adapted from [45].

Figure 4 presents a spatial pattern of mean temperature variation in Russia for the period 1976–2012 for the year as a whole and separately in each of the four seasons.

**Figure 5:**
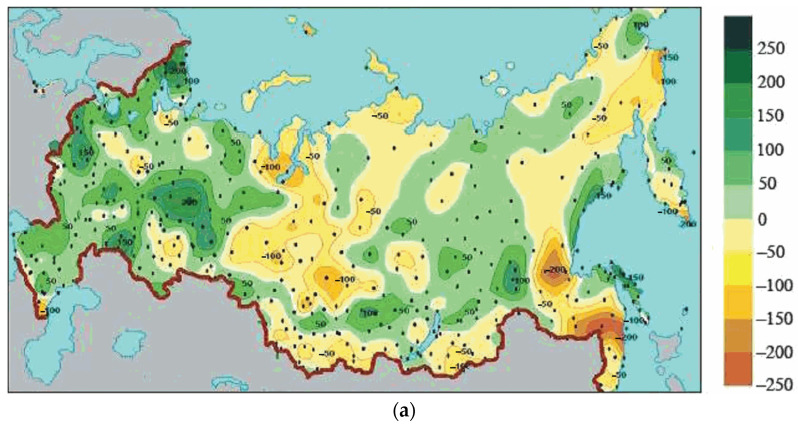

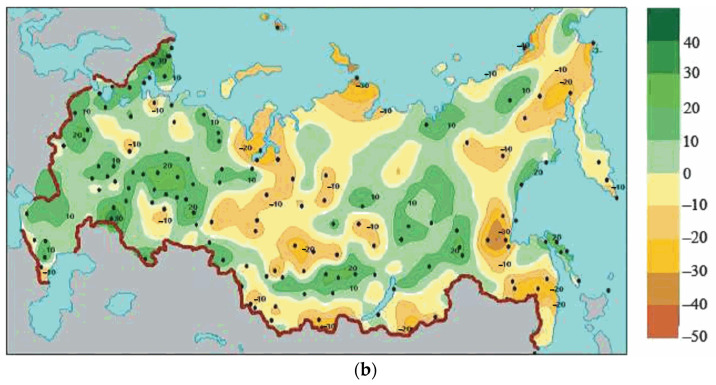
Spatial pattern of annual precipitation changes during 1936–2010 in Russia: (**a**) over the last 75 years, mm; (**b**) % of the mean value calculated for 1961–1990. Adapted from [45].

Figure 5 presents a spatial pattern of annual precipitation changes during 1936–2010 in Russia in mm and % of the mean value in 1961–1990. Summer temperature dynamics from Figure 4 and precipitations records from Figure 5 at 60 parallel were used for the comparison between European and Asian parts of Russia.

**Figure 6:**
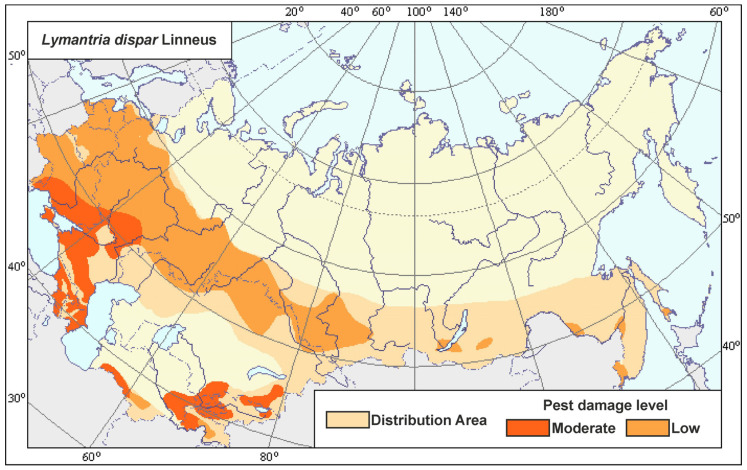
Range of harmfulness of the spongy moth *Lymantria dispar* (L.) [47].

Using the spatial data on *L. dispar* population distribution from both field research and literature publications, we performed a meta-analysis which allows us to combine information from such different sources with spatial data on climate changes along reference border line 60° N (see Figure 5 and Figure 6) in European and Asian parts of the Russian Federation. It is known that climate changes in the Russian Federation are more pronounced than world averages [45], which is why these factors should be considered for the analysis of *L. dispar* population distribution borders.

## 3. Results

### 3.1. Historical Records of L. dispar Observations

At the end of the 19th century, large-scale spongy moth reproduction outbreaks began to be noted in more northern regions—Tula, Moscow, Vladimir, Yaroslavl governorates, and Mari ASSR (now the Republic of Mari El). In the 1890s and in the 1930s and 1950s, outbreaks occurred over several million hectares [31,32,33,34,35,36]. After the 1960s, outbreaks were recorded annually throughout the rest of the 20th century.

**Figure 7:**
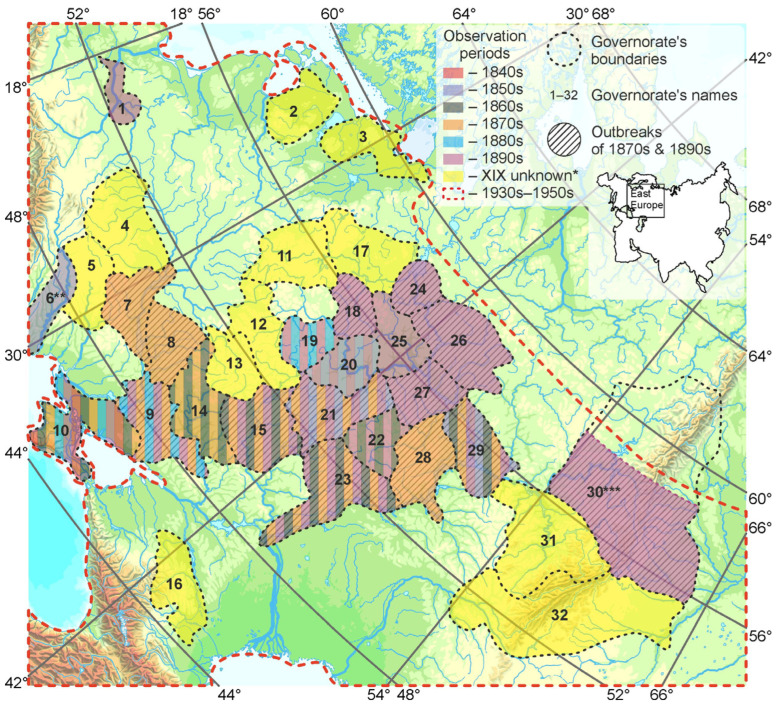
Mass reproduction outbreaks of the spongy moth according to publication materials of the 19th and first decade of the 20th century [22,24,25,26,27,28,29,30,31,32,33,34,35,36,37,38,39,40]. *—Mentioned in [22] without references. 1—Warsaw g., 2—Livonia g., 3—Saint Petersburg g., 4—Volyn g., 5—Podolia g., 6 **—Bessarabia g. (part), 7—Kiev g., 8—Poltava g., 9—Yekaterinoslav g., 10—Taurida g., 11—Smolensk g., 12—Oryol g., 13—Kursk g., 14—Kharkov g., 15—Voronezh g., 16—Stavropol g., 17—Tver g., 18—Moscow g., 19—Tula g., 20—Ryazan g., 21—Tambov g., 22—Penza g., 23—Saratov g., 24—Yaroslavl g., 25—Vladimir g., 26—Kostroma g., 27—Nizhny Novgorod g., 28—Simbirsk g., 29—Kazan g., 30 ***—Perm g. (with distribution boundary of *L. dispar*), 31—Ufa g., 32—Orenburg g.

Reports of the reproduction of this pest came from the central and southern regions of the European part of the Soviet Union, Siberia, and the Far East [35,36,37]. Currently, the range of harmfulness of the spongy moth also covers this entire territory [45,46,47,48]. In the agroeconomic atlas [47], the northwestern regions (Novgorod, Pskov, and the southern part of the Leningrad oblast) are shown as zones of low harmfulness, i.e., regions where an increase in population density of this pest can be expected (Figure 6). However, our analysis of the literature (Figure 7) and sanitary surveys in the 21st century did not reveal a single fact about any significant increase in the abundance of the spongy moth in these territories [6,49,50].

The northern boundary of the spongy moth range in the European part of Russia is associated with the oak range. In the mid-20th century, O.G. Kellus [38] and Ya.V. Chugunin [37] drew the northern boundary of this pest′s range along a line connecting St. Petersburg, Vologda, Kirov, and Perm. N.M. Kulagin noted that the spongy moth was often confused with the nun moth, so he personally verified the species identification using collections. The northernmost regions where the spongy moth was found included Kostroma, Tver, Moscow, Yaroslavl, and Livonia (southern part of modern Estonia and northern Latvia) governorates [22], St. Petersburg governorate [39], including the Oranienbaum area [22], as well as the Pskov oblast, the area of Strugi Krasnye village [40] and Novgorod oblast, and the Novgorod district without locality specification [48,51]; for the Leningrad oblast, one specimen from the Beloostrov area (southwestern part of the Karelian Isthmus) was noted [52]. In the species list of living organisms in the Leningrad oblast [53], the spongy moth is listed as an endangered species. In the reference book Forest Pests [54], the northern boundary in the European part of Russia was drawn along the line St. Petersburg–Vologda–Perm.

According to recently published data, the northern boundary of the spongy moth range covers the Novgorod, Pskov, and Leningrad oblasts and passes through the Arkhangelsk oblast and the Komi Republic [55]. Several findings have been documented in the southern part of the Komi Republic: Letka village 59°36′24.1″ N 49°25′05.1″ E (1982, two specimens); Shutrem village 60°57′09.7″ N 49°26′53.5″ E (2000, two specimens); and Vizinga village 61°04′34.3″ N 50°04′43.0″ E (2001, one specimen) [56]. In the EPPO and GBIF databases, there are records of spongy moth findings in Estonia. In addition, the GBIF database shows findings of this pest on the southern border of Finland in close proximity to the Karelian Isthmus [57]. In the Asian part of the range, recent studies have shown that the spongy moth reaches the 61st parallel [23], while on the North American continent, it reaches approximately the 49th parallel [23,58].

The staff of the Department of Forest Protection, Wood Science and Game Management of SPbSFTU have been monitoring the state of pest populations in the territory of St. Petersburg and surrounding areas for many years. Moths, caterpillars, or egg masses of the spongy moth have never been recorded [49,50,59]. A.L. Lvovsky conducted monitoring of the Lepidoptera fauna in the territory of St. Petersburg for many years. The main part of the collections was created in 1975–1989. The spongy moth was also not recorded by him [60].

### 3.2. Current Monitoring of the Northern Range Border of L. dispar in Europe

In all positive traps where moths were captured, instead of *L. dispar*, only the closely related *L. monacha* was captured. The correspondence of male genitalia to the nun moth species was verified to the reference specimen (Figure 3).

At capture locality 1, due to material decomposition, species identification was possible only for 122 specimens in 2023 and 35 specimens in 2024. The total number of captured individuals represents a minimum estimate of the possible number of moths caught. In all captures, and even in 2024 when pheromone only for spongy moth was used, only nun moth males were present.

It is quite natural that the greatest number of nun moths was collected by traps on the Karelian Isthmus at localities 1 and 2, where pine forests predominate. In the Pskov oblast (locality 6), all 85 specimens of nun moth were collected in a trap installed in a pine forest. In the trap installed in the garden, no specimens of nun moth were caught in either 2023 or 2024.

### 3.3. Comparison of Climate Dynamics in Northern Europe and Northern Asia

If we examine the 60° N latitude reference line in Figure 4c, we can see that the Asian part of Russia warming, as indicated by temperature increases, was much more significant compared to the European part. In the Asian part, the increase was as high as 0.8 °C per decade and even more to the north of 60° N latitude, up to 1.0 °C per decade, whereas in the European part, it was only 0.4 °C per decade or lower in the northern part. Thus, the rate of summer temperature increase is twice as high in Asia as in Europe (Table 2).

We can also observe that the rate of drought occurrence in Asia and Europe differs significantly at 60° N latitude: in the European part, the climate has a trend to be more wet, while in central Asia it is vice versa—it tends to be drier (Table 2).

Both climatic factor changes create more favorable environmental conditions for the distribution of *L. dispar* to the north from the 60° N latitude line in the continental part of Asia.

## 4. Discussion

Combining our literature data analysis and current observations in the 2020s, we demonstrate that although earlier records of *L. dispar* were occasionally reported near 60° N latitude in the European part of Russia, we cannot detect this species using highly sensitive methods of monitoring today. Mapping of the *L. dispar* range [47] demonstrates that the outbreak area of *L.dispar* reaches 60° N latitude in the European part. Our review of the data demonstrates that this phenomenon is unlikely or occurs rarely. For example, the collection of the Zoological Institute of RAS also contains four specimens (all males) from three different localities: Old Peterhof (two males, 25 July 1955), Razliv village in Luzhsky district (one male, 8 August 1962, moth reared from caterpillar), and Beloostrov (one male, 4 August 1980). No specimens from the Novgorod and Pskov oblasts were found in the collection. This suggests that a residual low density of *L. dispar* population inhabited areas near 60° N latitude in the last century, which might be related to spatial pulsation of the range boundary [61]. However, at present, we cannot detect even traces of this population in the northeastern European part.

K. Mikkola [62,63] explains the appearance of spongy moth in Finland by the movement of a significant number of moths in air currents. A.I. Vorontsov [64] also noted the appearance of migration foci of spongy moths in the Leningrad oblast and believed that these were random introductions of moths. However, even experimental studies on moths of Asian subspecies *L. dispar asiatica* and *L. dispar japonica*, whose females exhibit maximum flight activity, showed that the maximum active flight distance is at most 10 km [65,66]. Over distances of several kilometers [2,35,63,67], and possibly tens of km [68], spongy moth caterpillars can disperse via ballooning. This may be an alternative way for populations with non-flying females to significantly shift the species′ range boundaries. Nevertheless, the validity of claims regarding mass accumulation of moths at the latitude of St. Petersburg remains a matter of debate.

Our previous study of the northern border of the *L. dispar* range in the Asian part demonstrated active northward movement of the studied pest [11], which contrasts with the current study in the European part. Our analysis of heat accumulation dynamics near 60° N latitude demonstrates that although the annual temperature rise is higher in the European part of Russia compared to continental Asia (Figure 4a), the rate of summer temperature increase is twice as high in continental Asia (Table 2). Summer temperatures are more important than winter temperatures for *L. dispar* for the following reasons. First, *L. dispar* is a highly adaptive species with a low supercooling point (−25 to −30 °C) [69,70,71,72]. Moreover, this species exhibits high behavioral adaptability, allowing it to overwinter even with winter temperatures below this threshold. In particular, *L. dispar* females lay egg masses below the snow cover level [71] or lay them on rocky outcrops that do not allow temperatures to drop below the supercooling point [72]. Both of these adaptations allow the species to tolerate winter atmospheric temperatures below their survival limit. Thus, comparing the reaction of northern populations of *L. dispar* in Asia and Europe demonstrates a greater response by Asian populations to summer temperature increases through northward expansion compared to European populations, which remain below 60° N latitude, even against the background of warming winters (Figure 4b).

Another potentially limiting factor may be the availability of food resources. On the one hand, the spongy moth, being a broadly polyphagous species, can adapt to feeding on less favorable plant species, including conifers, while maintaining relatively high survival and fecundity [73,74]. On the other hand, in studies of the chemical profiles of leaves of some host plants in the latitudinal gradient in the Asian part of the spongy moth range, we also found no evidence of increased constitutive resistance to insects of plants with increasing expansion [75]. However, if we consider the temporal context of a factor important for plant resistance—precipitation amount—we see opposite patterns in the European and continental Asian parts (Table 2): in continental Asia, we observe slight aridification, while in the European part, precipitation has increased.

Precipitation dynamics also demonstrate that drought (an important factor for outbreak formation [76,77]) is a more limiting factor in Central Asia, where general aridification is occurring compared to the European part of the range (Figure 5). The water stress hypothesis postulates that drought decreases the resistance of host plants against herbivores [76,77]. Recent reviews indicate that severe drought is more important for generalist herbivores (like *L. dispar*), while moderate drought is important for the performance of specialized herbivores [78]. Thus, the continental Asian part of the *L. dispar* range is more predisposed to population increases than the European part of the range. Ballooning of young *L. dispar* larvae in a northeasterly direction during outbreaks [68] increases the effectiveness of the dispersal of *L. dispar* northward in continental Asia.

Another reason for this contrasting pattern of *L. dispar* range expansion between Eastern Europe and the continental Asian parts of the range may be the recent trend of increasing biodiversity in the northeastern part of the European continent [79]. It is known that the ability to rapidly increase population numbers is favored by monoculturization at the previous trophic level [80], i.e., host plants for *L. dispar*. On the other hand, the closely related but more specialized species *L. monacha* is expanding its distribution in the studied region (data from our study, [81]) under the same conditions of increasing biodiversity. Perhaps opposite trends in drought patterns between European and Asian parts drive insect populations with different levels of specialization (i.e., *L. dispar* vs. *L. monacha*) in different directions.

Finally, the population genetic structure (i.e., internal factors) demonstrates relative homogeneity for spongy moth populations close to the studied regions in both Europe and Continental Asia [82] and very homogeneous populations in Asia (Western Siberia) [68].

Some studies claim that the temperature threshold for the development of different life cycle stages of spongy moth represents the main factor limiting the northward expansion of this species. In particular, V.I. Ponomarev et al. [23] reviewed such studies. In the study by Titkina et al. [83], a climate-driven model of the spongy moth range in 1981–2010 compared to the period 1951–1980 was presented. According to this model, the northern boundary of the range should pass through the southern part of the Republic of Karelia. Indeed, a significant increase in cumulative effective temperatures in the Leningrad oblast and Karelia has occurred [84]. However, no spongy moths have been reported in Karelia or in the Leningrad oblast in the last 45 years, although lepidopterologists have been actively working in this area. The most recent record dates to the 1980s [52]. Apparently, thermal availability alone is a necessary but not sufficient factor for sustained development of spongy moth populations.

## 5. Conclusions

Thus, despite unambiguous forecasts from predictive models of northward advancement of range boundaries further north, the actual response of the range boundaries of an important invasive species, the spongy moth, varies in different parts of the range: while in the Asian part of the range, northern range boundary advancement corresponds to the predictions, in the European part, it does not. Our integrated analysis of 200 years of population dynamics reveals the opposite pattern; the spongy moth has been encountered less frequently in recent years. We demonstrate strong associations between long-term summer temperature dynamics, precipitation patterns, and population movements. Interestingly, a decrease in population density has also been noted in northern Europe for some other species of open-living phyllophages [6,9,50]. The causes of this phenomenon require further detailed investigation. Thus, our main finding is that the effects of climate warming differ significantly even among different populations of the same species.

## Figures and Tables

**Table 1:** Description of locality conditions where *L. dispar* monitoring was conducted.

Locality	No *	Coordinates	Locality Description
Leningrad oblast	1	60°34′51.9″ N 30°31′38.0″ E	Pine–bilberry/lichen forest, 110–140 years, medium density; mixed plantation adjacent to the site: birch, willow, alder
	2	60°14′28.3″ N 29°37′47.1″ E	Garden: apple, plum, currant
	3	59°59′39.1″ N 30°20′16.8″ E	Arboretum: mixed deciduous plantations, including fruit trees, and various tree species
	4	59°53′04.8″ N 29°54′29.0″ E	Old apple orchard
	5	59°12′25.4″ N 29°58′47.1″ E	Mixed forest: birch, alder, spruce/Garden: apple trees, plums, cherry
Pskov oblast	6	58°28′48.8″ N 27°50′17.2″ E	Pine forest, 70–100 years/Garden: apple trees, birches
	7	58°18′08.3″ N 30°31′16.7″ E	Garden: apple trees, plums, cherries
Novgorod oblast	8	58°32′04.4″ N 35°01′10.0″ E	Mixed forest: alder, birch, pine

* In 2023, pheromones for both spongy moth and nun moth were used simultaneously in each trap; in 2024, pheromones only for spongy moth were used.

**Table 2:** Temperature and precipitation changes at reference latitude line 60° N for European and Asian parts of the Russian Federation.

Climate Parameter Change	Part of Russian Federation on Reference Latitude Line 60° N		
	European	Asian	Asian/European
Temperature, °C per decade	0.4	0.8	2
Precipitation, mm	+0–50	−50–100	2
Precipitation, %	+0–10	−10–20	2

## Data Availability

The original contributions presented in this study are included in the article. Further inquiries can be directed to the corresponding authors.
